# Trends in Cardiovascular Mortality in Patients With Chronic Kidney Disease From 1999 to 2020: A Retrospective Study in the United States

**DOI:** 10.1002/clc.70174

**Published:** 2025-07-31

**Authors:** Eeman Ahmad, Shoaib Ahmad, Azka Naeem, Shahzaib Ahmed, Maryam Shehzad, Umar Akram, Hamza Ashraf, Obaid Ur Rehman, Irfan Ullah, Raheel Ahmed, Chadi Alraies, Gregg C. Fonarow

**Affiliations:** ^1^ Department of Medicine Fatima Memorial Hospital College of Medicine and Dentistry Lahore Pakistan; ^2^ St. Joseph Hospital and Medical Center Phoenix Arizona USA; ^3^ Department of Internal Medicine Maimonides medical centre Brooklyn NY USA; ^4^ Department of Medicine Dow University of Health Sciences Karachi Pakistan; ^5^ Department of Medicine Allama Iqbal Medical College Lahore Pakistan; ^6^ Medstar Health Georgetown University Baltimore USA; ^7^ Department of Internal Medicine Khyber Teaching Hospital Peshawar Pakistan; ^8^ National Heart and Lung Institute Imperial College London London UK; ^9^ Department of Cardiology Detroit Medical Center Detroit MI USA; ^10^ Division of Cardiology University of California Los Angeles CA USA

**Keywords:** cardiovascular disorders, chronic kidney disease, mortality

## Abstract

**Background:**

Chronic kidney disease (CKD) may be associated with fatal cardiovascular diseases (CVDs). We aim to identify CVD‐related mortality trends in patients with CKD in the US, examining the variation by sex, race, and region, and compare them to CVD‐related mortality trends in general.

**Methods:**

The CDC‐WONDER database was used to obtain age‐adjusted mortality rates (AAMRs) per 100,000 population. Annual percent change (APC) and average APC (AAPC) in these rates were calculated using Joinpoint regression and comparisons were done using pairwise comparison.

**Results:**

From 1999 to 2020, a total of 605,384 CVD‐related deaths were observed in patients with CKD. The AAMR was almost double in males (11.0) than females (6.3). NH (Non‐Hispanic) Blacks or African Americans displayed the highest overall AAMR while NH Asians or Pacific Islanders displayed the lowest. AAMRs also varied substantially by region (Midwest: 8.8; West: 8.6; South: 8.0; Northeast: 7.3). States with the highest AAMR was the District of Columbia. Nonmetropolitan regions exhibited a slightly higher AAMR (8.6) than metropolitan regions (8.1). The AAPC for CVD‐related deaths in patients with CKD differed significantly from that of the general population for the entire cohort, across both sexes, as well as among NH Whites, NH Black or African Americans, and Hispanics or Latinos. Regional differences were also observed in the Midwest, Northeast, and West.

**Conclusion:**

Significant differences in CVD‐related deaths in patients with CKD were observed. These high‐risk groups should be the point of focus for targeted interventions to reduce CVD‐related mortality in CKD patients.

## Graphical abstract

This study highlights significant disparities in CVD‐related mortality among individuals with CKD with various racial, gender, region, and urbanization‐based stratifications. Targeted efforts should focus on improving healthcare access, preventive strategies, and tailored interventions based on high‐risk groups to reduce disparities in CVD‐related mortality among CKD patients.

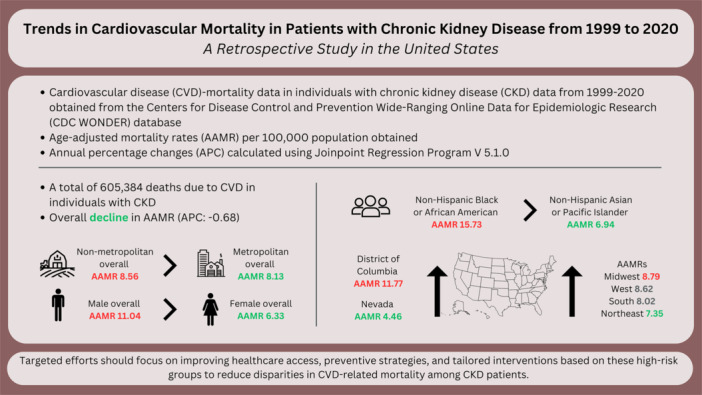

## 1 Introduction

Chronic kidney disease (CKD) has emerged as a significant global public health concern, with an estimated worldwide prevalence of 13.4% [1, 2]. In the United States alone, the Centers for Disease Control and Prevention (CDC) reports that approximately 1 in 7 adults, or about 35.5 million individuals (14% of the adult population), are affected by CKD [3]. The condition places a substantial medical and financial burden on societies and healthcare systems due to its strong association with increased mortality, cardiovascular disease (CVD), and progression to end‐stage kidney disease [4, 5].

CVD remains the leading cause of death among individuals with CKD [6]. Elevated cardiovascular risk is evident even in the early stages of CKD and becomes more pronounced as the disease advances [7]. This increased risk is driven by both traditional cardiovascular risk factors—such as hypertension, diabetes, and dyslipidemia—and CKD‐specific factors, including uremic toxins, inflammation, and vascular calcification [7, 8]. Hypertension is a critical contributor to cardiac damage at all CKD stages, while anemia plays a role in the development of left ventricular hypertrophy [9]. Furthermore, calcium‐phosphate imbalances and emerging risk factors for atherosclerosis further compound cardiovascular risk in this patient population [9].

Despite the well‐established link between CKD and CVD, cardiovascular disease frequently remains underdiagnosed and inadequately managed in these high‐risk patients [8]. This study aims to analyze trends in CVD‐related mortality among CKD patients in the U.S. over the past two decades, offering a comprehensive evaluation of demographic differences across age, gender, race, and geographic regions. Understanding these trends is crucial for identifying at‐risk populations and informing targeted preventive and therapeutic strategies.

## 2 Methods

### 2.1 Study Design

We analyzed trends in mortality related to CVD among patients with CKD in the US from 1999 to 2020, using data from the Centers for Disease Control and Prevention's Wide‐ranging ONline Data for Epidemiologic Research (CDC WONDER) Database [10]. The comparison cohort comprised all individuals who died with CVD listed as the primary cause of death, regardless of contributing causes, from 1999 to 2020. CDC WONDER utilizes death certificate data to present both underlying and multiple causes of death, along with demographic information.

We used the International Classification of Diseases, 10th Revision (ICD‐10) [11] codes I00‐I99 for CVD [12] and N18 for CKD [13] to identify death certificates for individuals with CVD as the underlying cause of death and CKD as the multiple cause of death.

We also stratified these deaths further by stages of CKD and extracted data for individual stages, namely end‐stage renal disease (ICD code N18.0), CKD stage 1 (ICD code N18.1), CKD stage 2 (ICD code N18.2), CKD stage 3 (ICD code N18.3), CKD stage 4 (ICD code N18.4), and CKD stage 5 (ICD code N18.5). These codes were used for multiple cause of death, while CVD remained the underlying cause of death.

ICD codes N18.1‐N18.5 were added to the CDC WONDER database in 2011. Therefore, stage‐specific data was not available for prior years. However, N18.0 (end‐stage renal disease) was used from 1999 to 2010 in the database, providing data equivalent to CKD stage 5.

This study utilized anonymized, publicly available data, so it did not require ethical approval from an Institutional Review Board (IRB). This study conformed to the Strengthening the Reporting of Observational Studies in Epidemiology (STROBE) guidelines [14].

### 2.2 Data Abstraction

Data was stratified by sex, race/ethnicity, census region, urbanization level, and state. Sex was categorized as male or female. Race and ethnicity groups included Non‐Hispanic (NH) Asian or Pacific Islander, NH White, NH American Indian or Alaska Native, NH Black or African American, and Hispanic or Latino. Census regions were divided into Northeast, Midwest, South, and West. Urbanization levels were determined based on the 2013 NCHS Urban‐Rural Classification Scheme for Counties [15] and categorized as urban (large central metro, large fringe metro, medium metro, and small metro) or rural (micropolitan and noncore areas).

### 2.3 Statistical Analysis

The crude mortality rates (CMR) per 100,000 individuals were calculated by dividing the number of cause‐specific deaths by the total population for the specified year. The age‐adjusted mortality rate (AAMR) was determined by applying age‐specific mortality rates to the age distribution of the USA 2000 standard population, providing a weighted average for fair comparisons across different populations or time periods. Mortality rate trends were examined using the Joinpoint Regression Program (version 5.1.0, National Cancer Institute). This tool applies serial permutation tests to detect repeated time trends and identifies up to one significant inflection point where the change rate shifts [16]. For each time segment, the annual percentage change (APC) was calculated, along with the associated 95% confidence interval (CI). We also performed a pairwise comparison with a comparison cohort of CVD as the primary cause of death using the same program to examine whether there were statistically significant differences in APCs between both cohorts across various stratifications. A *p*‐value of < 0.05 was considered to be significant.

## 3 Results

**Figure 1:**
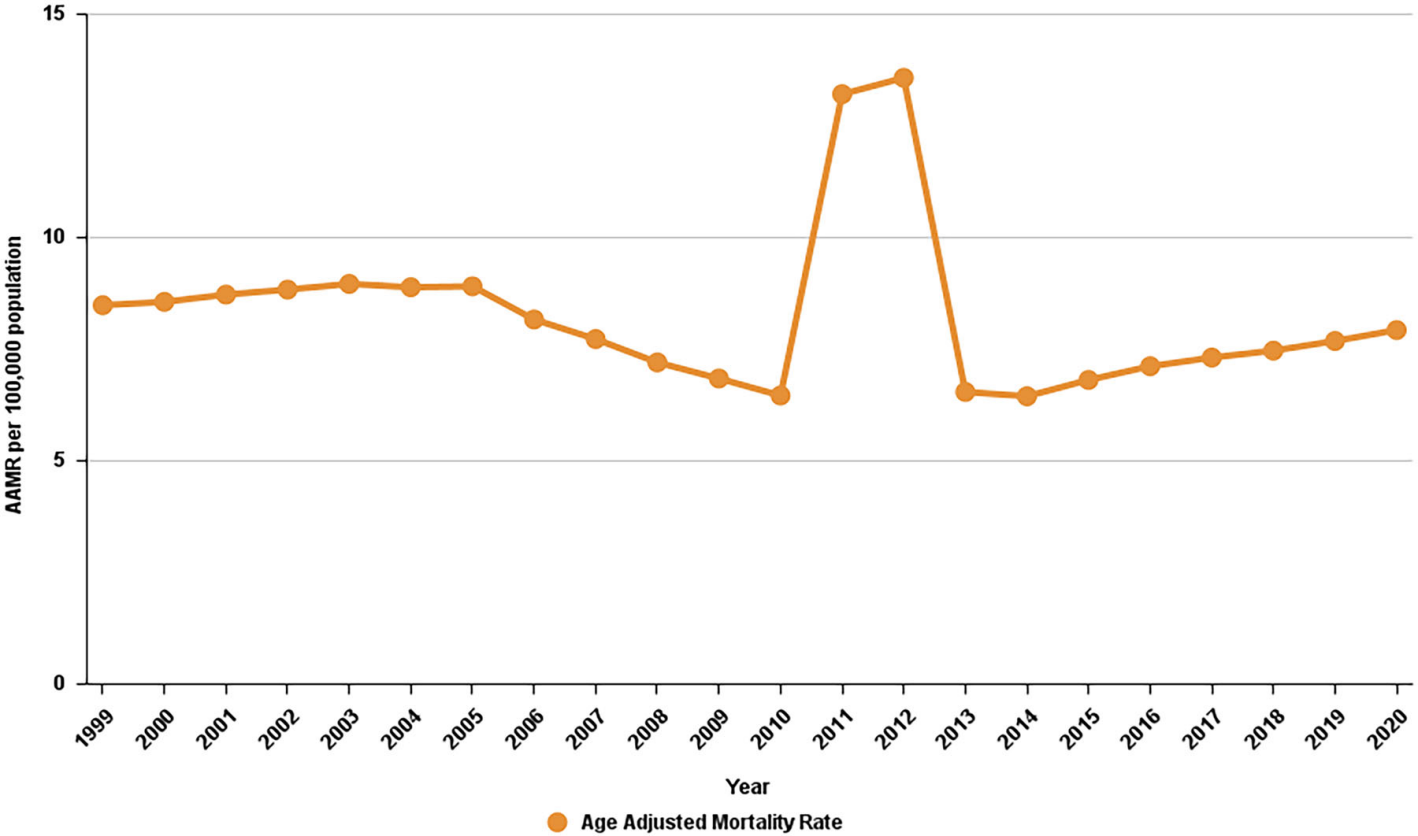
Trends in cardiovascular‐related mortality in patients with chronic kidney disease in the United States from 1999 to 2020.

Over the study period, a total of 605,384 CVD‐related deaths were reported among patients with CKD ***(*** Table 1 ***)***. Between 1999 and 2020, the AAMR for this group showed a slight decline, from 8.5 to 7.9 per 100,000 population, with an average annual percentage change (AAPC) of −0.68% (95% CI: −2.28 to 0.94; *p* = 0.39), indicating that the AAMR remained relatively stable over time. In the general population, there were 18,783,791 CVD‐related deaths during the same period. The AAMR decreased significantly, from 350.8 in 1999 to 224.4 in 2020, with an AAPC of −2.32% (95% CI: −2.53 to −2.12; *p* < 0.000001) (Tables 2 and 3, Figure 1).

**Table 1:** Demographic characteristics of deaths due to cardiovascular disorders in patients with chronic kidney disease in the United States from 1999 to 2020.

Variable	Deaths	Population	AAMR (95% CI)
Overall	605,384	6,746,356,647	8.20 (8.18−8.22)
**Sex**			
Female	278,547	3,429,003,804	6.33 (6.30−6.35)
Male	326,837	3,317,352,843	11.04 (11.01−11.08)
**Race/Ethnicity**			
Hispanic or Latino	41,500	1,077,280,338	7.79 (7.71−7.87)
NH American Indian or Alaska Native	3776	56,210,240	9.85 (9.52−10.18)
NH Asian or Pacific Islander	18,444	354,753,001	6.94 (6.84−7.04)
NH Black or African American	102,234	863,931,810	15.37 (15.27−15.47)
NH White	438,206	4,394,181,258	7.35 (7.33−7.37)
**Census Region**			
Northeast	108,940	1,212,994,922	7.34 (7.30–7.39)
Midwest	147,969	1,466,121,214	8.80 (8.75–8.84)
South	213,482	2,497,818,081	8.02 (7.99–8.06)
West	134,993	1,569,422,430	8.62 (8.57–8.67)
**Urbanization**			
Urban	493,892	5,739,475,649	8.12 (8.10–8.15)
Rural	111,492	1,006,871,652	8.57 (8.52–8.62)

Abbreviations: AAMR, age‐adjusted mortality rate; CI, confidence interval.

**Table 2:** Annual percentage changes (APCs) and average annual percentage changes (AAPCs) in cardiovascular disorders in patients with chronic kidney disease in the USA from 1999 to 2020.

Variable	Trend segment	Lower endpoint	Upper endpoint	APC (95% CI)	AAPC (95% CI)	*p* value
Entire Cohort	1	1999	2020	−0.68 (−2.23 to 1.00)	−0.68 (−2.23 to 1.00)	0.435913
**Sex**						
Female	1	1999	2020	−0.81 (−2.47 to 0.95)	−0.81 (−2.47 to 0.95)	0.371126
Male	1	1999	2020	−0.80 (−2.25 to 0.80)	−0.80 (−2.25 to 0.80)	0.339532
**Race**						
NH American Indian or Alaska Native	1	1999	2020	−1.84* (−3.35 to −0.15)	−1.84* (−3.35 to −0.15)	0.034393
NH Asian or Pacific Islander	1	1999	2009	−4.86* (−15.73 to −1.42)	−3.16* (−4.77 to −1.51)	0.002000
	2	2009	2012	21.60* (3.92 to 34.71)		
	3	2012	2015	−29.45* (−36.80 to −16.32)		
	4	2015	2020	5.82 (−1.70 to 28.67)		
NH Black or African American	1	1999	2020	−2.59* (−4.39 to −0.74)	−2.59* (−4.39 to −0.74)	0.009598
NH White	1	1999	2020	−0.04 (−1.51 to 1.55)	−0.04 (−1.51 to 1.55)	0.991802
Hispanic or Latino	1	1999	2020	−2.71 (−4.43 to −0.72)	−2.71 (−4.43 to −0.72)	0.007199
**Census Region**						
Northeast	1	1999	2020	−1.09 (−2.44 to 0.31)	−1.09 (−2.44 to 0.31)	0.122775
Midwest	1	1999	2020	−0.02 (−1.51 to 1.59)	−0.02 (−1.51 to 1.59)	0.974605
South	1	1999	2020	−0.88 (−2.41 to 0.78)	−0.88 (−2.41 to 0.78)	0.319536
West	1	1999	2020	−0.77 (−2.60 to 1.26)	−0.77 (−2.60 to 1.26)	0.465907
**Urbanization**						
Urban	1	1999	2020	−0.81 (−2.37 to 0.88)	−0.81 (−2.37 to 0.88)	0.349930
Rural	1	1999	2020	−0.05 (−1.48 to 1.46)	−0.05 (−1.48 to 1.46)	0.976205

Abbreviations: APC, annual percent change; AAPC, average annual percent change; CI, confidence interval.

* Indicates that the APC or AAPC is significantly different from zero at the alpha = 0.05 level.

**Table 3:** Pairwise comparison between cardiovascular disorders‐related mortality rates in patients with chronic kidney disease and overall cardiovascular disorders‐related mortality rates in the USA from 1999 to 2020.

Variable	Cardiovascular disorders‐related mortality rates in patients with chronic kidney disease		Overall cardiovascular disorders‐related Mortality		*p* value for AAPC comparison
	AAPC (95%CI)	*p* value	AAPC (95%CI)	*p* value	
Overall	−0.68 (−2.27 to 0.94)	0.390149	−2.33* (−2.53 to −2.12)	< 0.000001	0.009556*
**Sex**					
Male	−0.80 (−2.29 to 0.72)	0.285450	−2.22* (−2.42 to −2.02)	< 0.000001	0.014889*
Female	−0.81 (−2.50 to 0.91)	0.335083	−2.38* (−2.87 to −1.89)	< 0.000001	0.014000*
**Race**					
Hispanic or Latino	−2.71* (−4.63 to −0.76)	0.009232	−2.30* (−2.79 to −1.80)	< 0.000001	0.031333*
NH American Indian or Alaska Native	−1.84* (−3.42 to −0.24)	0.026534	−1.80* (−2.20 to −1.40)	< 0.000001	0.344667
NH White	−0.04 (−1.54 to 1.48)	0.957480	−2.14* (−2.54 to −1.73)	< 0.000001	0.003778*
NH Black or African American	−2.59* (−4.33 to −0.82)	0.006494	−1.81* (−2.03 to −1.59)	< 0.000001	0.041333*
NH Asian or Pacific Islander	−3.16* (−5.55 to −0.72)	0.011554	−2.49* (−2.81 to −2.16)	< 0.000001	0.055556
**Census Region**					
Northeast	−1.09 (−2.51 to 0.34)	0.126955	−2.53* (−2.73 to −2.32)	< 0.000001	0.012667*
Midwest	−0.0 (−1.54 to 1.52)	0.978087	−2.03* (−2.37 to −1.68)	< 0.000001	0.013556*
South	−0.88 (−2.41 to 0.67)	0.246903	−2.15* (−2.50 to −1.80)	< 0.000001	0.056000
West	−0.77 (−2.72 to 1.22)	0.427886	−2.33* (−2.77 to −1.88)	< 0.000001	0.004222*
**Urbanization**					
Urban	−0.81 (−2.43 to 0.85)	0.319167	−2.40* (−2.61 to −2.19)	< 0.000001	0.009778
Rural	−0.05 (−1.51 to 1.42)	0.938601	−1.90* (−2.10 to −1.71)	< 0.000001	0.011778

CVD‐mortality in patients with CKD stages 2 and 5 decreased from 2011 to 2020 (AAPC CKD stage 2: −4.86, stage 5: −7.70), and increased for patients with CKD stages 3 and 4 (AAPC CKD stage 3: 10.68, stage 4: 6.66). These codes were added to the database in 2011, and thus mortality data for 1999 to 2010 stratified by CKD stages were not available. However, data for end‐stage renal disease was available, and a decrease in CVD‐mortality was seen for these individuals (AAPC: −0.87) (Supporting Table S1).

### 3.1 Trends by Sex

Regarding CVD mortality among CKD patients, females accounted for 46% of the deaths (*n* = 278,547), while males accounted for 54% (*n* = 326,837). Males had a considerably higher AAMR for CVD and CKD‐related deaths compared to females (males: 11.04; females: 6.33). For males, the AAMR decreased slightly over the study period, from 11.54 in 1999 to 10.54 in 2020, with an AAPC of −0.79% (95% CI: −2.29 to 0.72; *p* = 0.285), indicating a relatively stable trend. Similarly, for females, the AAMR declined marginally from 6.63 in 1999 to 6.05 in 2020, with an AAPC of −0.81% (95% CI: −2.49 to 0.91; *p* = 0.335), but the trend also remained stable throughout the study period (Tables 1 and 2).

**Figure 2:**
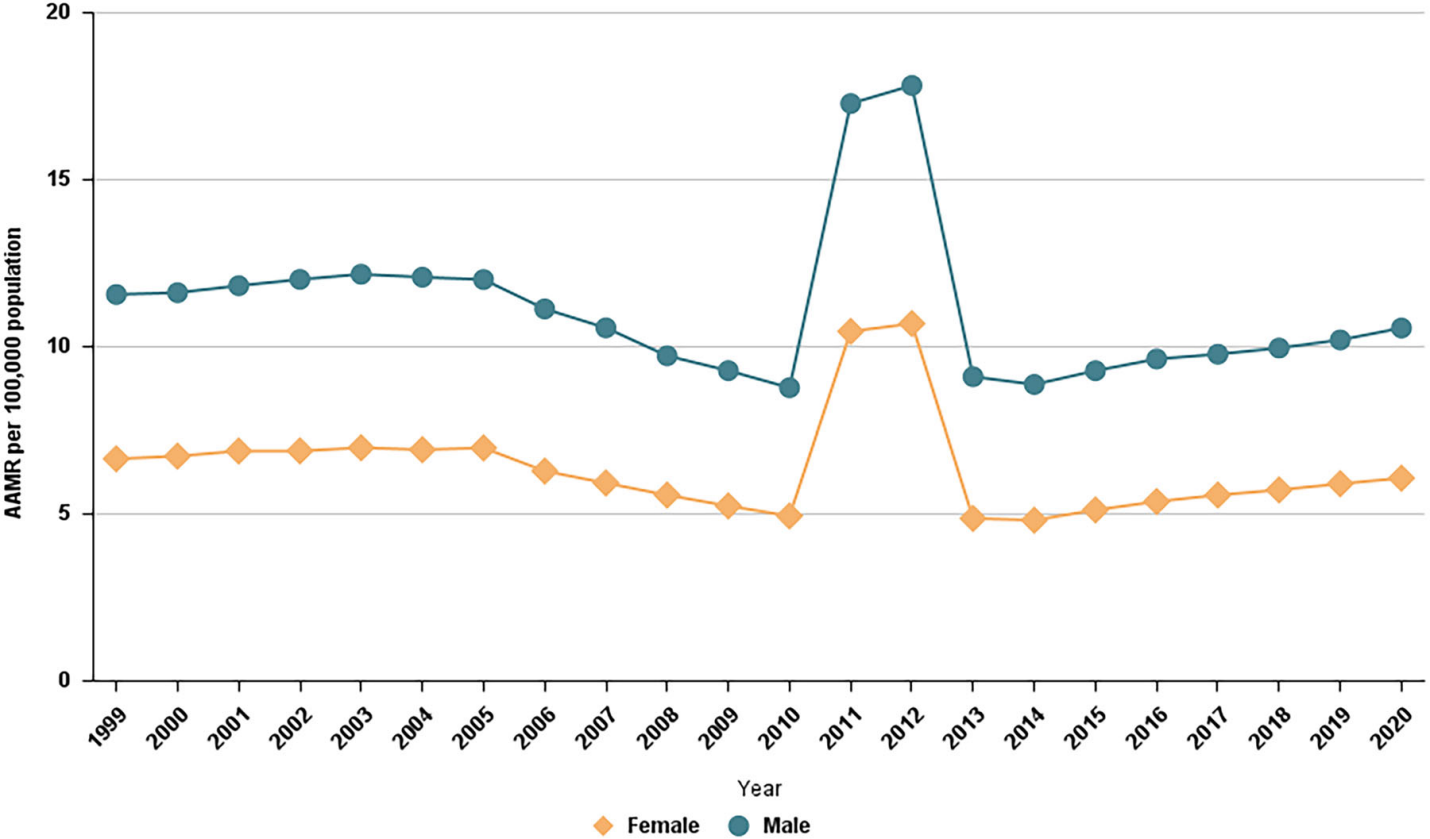
Trends in cardiovascular‐related mortality in patients with chronic kidney disease stratified by gender in the United States from 1999 to 2020.

Regarding CVD‐related mortality, females accounted for 51.5% of the deaths (*n* = 9,580,318), while males accounted for 48.9% (*n* = 9,203,473). Males exhibited a significantly higher AAMR for CVD‐related deaths compared to females (males: 303.73; females: 214.33). For males, the CVD‐related AAMR decreased steeply over the study period, declining from 420.5 in 1999 to 273.5 in 2020, with an AAPC of −2.22% (95% CI: −2.42 to −2.02; *p* < 0.000001), reflecting a downward trend. Similarly, females experienced a decline in AAMR from 297.9 in 1999 to 183.1 in 2020, with an AAPC of −2.38% (95% CI: −2.87 to −2.87; *p* < 0.000001), indicating a notable downward trend over the study period (Tables 2 and 3, Figure 2).

### 3.2 Trends by Race

Over the study period, NH White individuals accounted for the majority of deaths with CVD as the primary cause and CKD as a contributing cause at 72.38%, followed by NH Black or African American individuals at 16.89%. Hispanic/Latino individuals comprised 6.86% of these deaths, while NH Asian or Pacific Islander individuals accounted for 3.05%. NH American Indian or Alaska Native individuals represented the smallest proportion, at 0.62%. With regard to AAMR, the NH Black or African American group had the highest AAMR at 15.37, followed by NH American Indian or Alaska Native at 9.85, Hispanic or Latino at 7.78, NH White at 7.35, and NH Asian or Pacific Islander at 6.94 (Tables 1 and 2).

**Figure 3:**
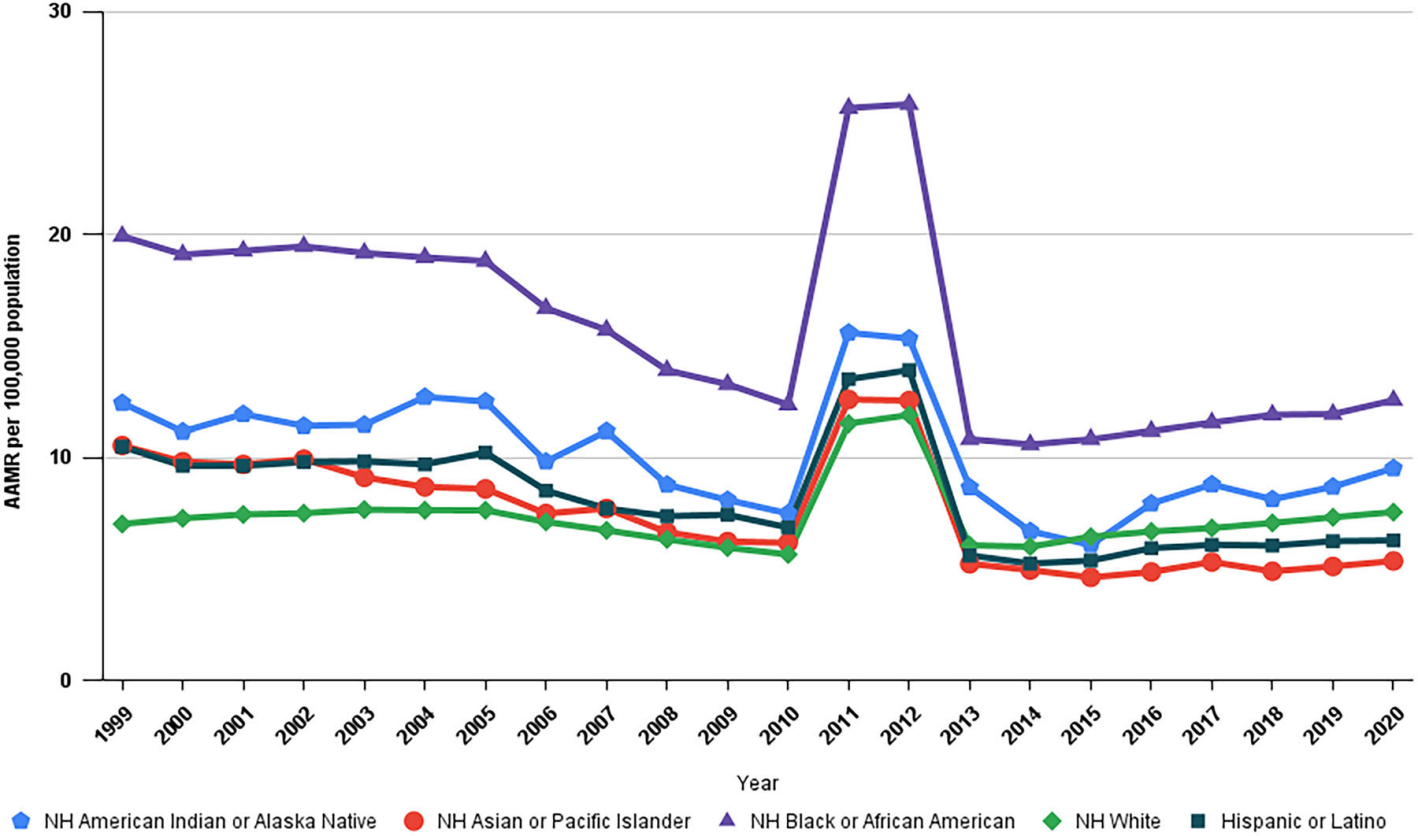
Trends in cardiovascular‐related mortality in patients with chronic kidney disease stratified by race/ethnicity in the United States from 1999 to 2020.

CKD and CVD‐related mortality showed a considerable decline across all racial groups in the United States over the study period. Among NH Black individuals, the AAMR decreased substantially from 19.93 in 1999 to 12.57 in 2020, with an AAPC of −2.59% (95% CI: −4.33 to −0.82; *p* = 0.0065). Similarly, NH American Indian or Alaska Native individuals experienced a reduction in AAMR, declining from 12.45 in 1999 to 9.54 in 2020, with an AAPC of −1.84% (95% CI: −3.42 to −0.24; *p* = 0.027). The Hispanic or Latino group also saw a decrease, with the AAMR falling from 10.53 in 1999 to 6.29 in 2020, corresponding to an AAPC of −2.72% (95% CI: −4.63 to −0.76; *p* = 0.009). In contrast, the NH White group showed relatively stable AAMR trends over the study period, with an AAPC of −0.0391% (95% CI: −1.53 to 1.48; *p* = 0.957). Lastly, the NH Asian or Pacific Islander group experienced a notable decline in AAMR, from 10.56 in 1999 to 5.36 in 2020, with an AAPC of −3.16% (95% CI: −5.55 to −0.72; *p* = 0.012) (Table 2, Figure 3).

With regard to CVD‐related mortality, all racial groups experienced a considerable decline over the study period. The NH Black or African American group had an AAPC of −1.82% (95% CI: −2.03 to −1.59; *p* < 0.000001), followed by the NH American Indian or Alaska Native group with an AAPC of −1.80% (95% CI: −2.20 to −1.40; *p* < 0.000001). The Hispanic or Latino group showed an AAPC of −2.29% (95% CI: −2.79 to −1.80; *p* < 0.000001), while the NH White group demonstrated an AAPC of −2.13% (95% CI: −2.54 to −1.73; *p* < 0.000001). Lastly, the NH Asian or Pacific Islander group exhibited the steepest decline, with an AAPC of −2.49% (95% CI: −2.81 to −2.16; *p* < 0.000001) (Table 2).

The trends in CKD mortality between the CVD population and the overall population within each racial and ethnic group (Hispanic and non‐Hispanic) differed significantly for the NH White population (*p* = 0.005) (Table 3).

### 3.3 Trends by Urbanization

Throughout the study period, nonmetropolitan areas consistently exhibited a higher average AAMR for deaths associated with CVD as the main cause and CKD as a secondary cause compared to metropolitan areas (nonmetropolitan: 8.56; metropolitan: 8.13). Regarding CVD and CKD‐related mortality in nonmetropolitan areas, the AAMR showed a slight increase from 7.81 in 1999 to 8.94 in 2020, remaining relatively unchanged over the study period (AAPC: −0.05%; 95% CI: −1.51 to 1.42; *p* = 0.938). In contrast, CVD‐related mortality in nonmetropolitan areas experienced a prominent decline, with the AAMR decreasing at an AAPC of −1.90% (95% CI: −2.10 to −1.71; *p* < 0.000001). Similarly, in metropolitan areas, CVD and CKD‐related mortality trends were relatively stable, with the AAMR decreasing slightly from 8.61 in 1999 to 7.73 in 2020 (AAPC: −0.80%; 95% CI: −2.43 to 0.85; *p* = 0.319). However, CVD‐related mortality in metropolitan areas showed a major decline, with the AAMR decreasing at an AAPC of −2.40% (95% CI: −2.61 to −2.19; *p* < 0.000001) (Tables 1 and 2, Supporting Figure S1).

In nonmetropolitan areas, CVD and CKD‐related mortality showed a significantly faster increase than CVD alone over the period 1999–2020 (*p* = 0.0092) (Table 3).

### 3.4 Trends by Census Region

The South recorded the highest percentage of deaths attributed to CVD as the primary cause and CKD as a contributing cause, accounting for 35.26% of the total deaths (213,482). This was followed by the Midwest at 24.44% (147,969), the West at 22.30% (134,993), and the Northeast with the lowest percentage at 17.99% (108,940). Regarding AAMR for deaths involving CVD and CKD, the Midwest had the highest rate at 8.79, followed by the West at 8.62, the South at 8.02, and the Northeast at 7.35. Regarding CVD‐related AAMR, the Southern region had the highest rate at 267.74, followed by the Midwest at 260.93, the Northeast at 249.05, and the West at 228.48 (Tables 1 and 2, Supporting Figure S2). AAPCs differed significantly from the general population for the Northeast (*p* = 0.0127), Midwest (*p* = 0.0136), and West (*p* = 0.0042) (Table 3).

### 3.5 Trends by States

The states with the highest AAMRs were the District of Columbia (11.77), West Virginia (11.03), North Dakota (10.73), Ohio (10.53), and California (10.29). In contrast, the states with the lowest AAMRs were New Mexico (5.31), Utah (5.23), and Nevada (4.46), reflecting a significant disparity in mortality rates across states (Supporting Table S2, Supporting Figure S3).

## 4 Discussion

Our database study highlights several key findings regarding trends for CVD‐related mortality in patients with CKD. A total of 605,384 CVD‐related deaths were reported among patients with CKD. The AAMRs showed a slight decline but remained relatively stable over time, and AAPC differed significantly from the general population. The AAMR was almost double in males than females in CKD cohort differing significantly from those without CKD. Highest overall AAMRs were displayed by NH Blacks or African Americans, while NH Asians or Pacific Islanders displayed the lowest. The AAMRs decreased for NH Blacks or African Americans, NH American Indians or Alaska Natives, and Hispanics or Latinos. The AAPC for NH Whites, NH Black or African Americans, and Hispanics or Latinos differed significantly from that of the general population. Midwest reported the highest AAMRs followed by the West, South, and Northeast. States with the highest AAMRs were the District of Columbia and West Virginia. The AAPC for CVD‐related deaths in patients with CKD differed significantly from those in the general population in the Midwest, Northeast, and West.

CKD is an independent CVD risk factor, and its prevalence has been rising steadily over the decades. Indeed, cardiovascular risk increases as the estimated glomerular filtration rate (eGFR) decreases [17]. Among CVD risk factors, hypertension is considered to be the strongest risk factor for CKD [18] A study reported an increase in hypertension‐related CVD deaths in the United States from 2012 [19], which could account for the high AAMR seen in 2011 and 2012 in comparison to other years in our study. In addition, national trends from 2011 to 2015 in another study show a rapid rise in cardiorenal syndrome and heart‐failure–related deaths during that period which could be the possible reason for this increase in AAMR in our study [20].

Traditional cardiovascular risk factors like hypertension, diabetes mellitus, and dyslipidemia are common in CKD and often difficult to control [21] However, patients with CKD also face additional, disease‐specific contributors to cardiovascular mortality, including vascular calcification, chronic inflammation, anemia, and abnormalities in calcium‐phosphate metabolism [22, 23] These pathophysiological processes lead to accelerated atherosclerosis and cardiac remodeling, placing CKD patients at particularly high risk for events such as sudden cardiac death and heart failure.

The interplay between heart and kidney dysfunction, often described as cardiorenal syndrome, further complicates management [24] Acute decompensation of one organ system frequently precipitates failure of the other, underscoring the need for integrated approaches to care.

Epidemiological studies show that CKD is more common in women compared to men [25], and gender significantly influences the pathogenesis of renal impairment. Notwithstanding these findings, women have been underrepresented in clinical studies of CKD and its complications. This underrepresentation motivated us to explore gender, racial, and geographic differences in trends for CVD death in patients with CKD.

Our study results indicated that the AAMR owing to CVD in patients with CKD was considerably high in males in comparison to females (males: 11.04; females: 6.33). This can be partly attributed to the protective influence of female sex hormones, which provide a survival advantage, as indicated by elevated mortality rates owing to CVD in males (303.73 for males vs. 214.33 for females). Despite alterations in the production of sex hormones, the inherent protective effect in females is a potential contributor to their comparatively low mortality rates compared with men [26]. This observation was validated by a large meta‐analysis of 48 studies involving 99,822 patients that also indicated a rise in cardiovascular mortality [27]. The fact that data from various geographic locations were included increases the generalizability of these findings. Moreover, the AAMR of both sexes showed a marginal decline over the study period, demonstrating an AAPC of −0.79% in males and −0.81% in females. The finding indicates that the overall trend in mortality rates has been more or less consistent.

The racial discrepancy analysis identified that the NH Black or African American population possessed the highest AAMR of 15.37. This is in line with research published in 2017 [28], where it was discovered that African American patients had a greater prevalence of coronary artery disease (CAD) and heart failure, especially in those with CKD. In addition, African Americans with CKD are also more likely to experience resistant and difficult‐to‐control hypertension [29], which is an independent risk factor for CVD mortality. Among the Hispanic or Latino population, the AAMR seen was 7.78. This is partially explained by the higher prevalence of coronary artery calcification [30] and the propensity of CKD to cause vascular calcification, including coronary artery calcification. In contrast, the NH White population AAMR was 7.35. Although CAD is less prevalent in white patients in general, elevated levels of inflammatory markers such as interleukin‐6 (IL‐6) [31] have been associated with high inflammation and subsequent CAD risk in this group.

For the NH Asian or Pacific Islander group, the AAMR was 6.94. It must be noted here that although it is strongly established that South Asians are at very high risk for CAD, owing to their atherogenic lipoprotein profiles and dysglycemia [32] pooling East Asians along with South Asians in a single Asian group can lower the combined AAMR, since East Asians tend to have fewer of these risk factors. Furthermore, genome‐wide association studies (GWAS) have identified approximately 300 loci associated with diabetes, approximately 500 loci associated with obesity‐related characteristics and other cardiometabolic traits, and approximately 48 loci with common single nucleotide polymorphisms (SNPs) associated with CAD, including those identified in South Asian populations [33, 34].

Over time, CKD and CVD mortality declined significantly in all of the ethnic groups examined, with AAPC of −2.59% in Blacks, −1.84% in American Indian or Alaska Natives, −2.72% in Hispanics, −0.0391% in Whites, and −3.16% in Asians. This decrease in mortality may stem from various factors such as advances in medical therapies and technological innovation, as well as national programs for increasing awareness of cardiovascular risk factors. Additionally, changes in acute cardiovascular care are likely to have played a role in reducing mortality. The advent of advanced revascularization modalities, the evolution of guideline‐directed drug therapies, and evolving standards of care for acute cardiovascular (CV) events have positively impacted patient care.

Geographic trends reveal that AAMR in nonmetropolitan regions is higher than in metropolitan regions (8.56 *vs.* 8.13). These findings are consistent with the results of a study conducted by Sekkarie et al. [35], which used county‐level data from 2021 and showed that high‐poverty rural areas located in the Southern region tend to have higher mortality rates. The Midwest had the highest AAMR for CVD and CKD deaths at 8.79 while the West had 8.62, the South had 8.02, and the Northeast had the lowest at 7.35.

Disparities in cardiovascular health outcomes are influenced by multiple factors, including socioeconomic status, healthcare accessibility, and structural barriers within the healthcare system. Additionally, variations in comorbidities, lifestyle choices, and other social determinants of health contribute to these inequities. Addressing these disparities requires a well‐defined, multi‐faceted approach. Key strategies include targeted public health interventions, expanded healthcare access for underserved populations, and tailored care for vulnerable groups. It is also essential to critically assess existing policies and interventions to determine their effectiveness in reducing racial, gender, and geographic disparities in cardiovascular health. Future efforts should focus on closing gaps in healthcare access, enhancing patient education, and improving treatment availability. Ensuring that preventive and management strategies for cardiovascular diseases benefit all populations equitably remains a crucial objective in advancing health equity.

The main strength of our study is the large sample size, which enables a comprehensive analysis of temporal trends and disparities across the entire U.S. population over the past two decades. However, several limitations should be considered when interpreting our findings. Firstly, our reliance on death certificate data may have resulted in an underestimation of actual cases [36, 37]. Secondly, we were unable to distinguish between different stages of CKD. Thirdly, the study did not account for the influence of comorbidities or socioeconomic factors. Fourthly, the database does not contain in‐depth clinical information such as laboratory findings, treatment history, or disease severity that is necessary to establish patient‐specific risk factors and outcomes. Fifthly, while the database provides state‐ and county‐level data, it lacks detailed socioeconomic indicators such as income, insurance, and access to healthcare, which are critical to examine health disparities. Lastly, it is important to note that we used overall CVD‐related mortality rates rather than proportional mortality rates, facilitating comparisons with existing literature.

## 5 Conclusion

In conclusion, this study highlights significant disparities in CVD‐related mortality among individuals with CKD with various racial, gender, region, and urbanization‐based stratifications. A significant difference in mortality trends was observed compared to that of the general population. Males exhibited two‐fold mortality rates than that of their counterparts. NH Blacks or African Americans and the Midwest had the highest mortality rates. Targeted efforts should focus on improving healthcare access, preventive strategies, and tailored interventions based on these high‐risk groups to reduce disparities in CVD‐related mortality among CKD patients.

## Author Contributions

**Eeman Ahmad:** conceptualization, project administration, formal analysis, writing – original draft, writing – review and editing, **Shoaib Ahmad:** Conceptualization, writing – original draft, writing – review and editing, **Azka Naeem:** conceptualization, writing – original draft, writing – review and editing, **Shahzaib Ahmed:** validation, writing – original draft, writing – review and editing, **Maryam Shehzad:** validation, writing – original draft, writing – review and editing, **Umar Akram:** validation, writing – original draft, writing – review and editing, **Hamza Ashraf:** validation, writing – review and editing, **Obaid Ur Rehman:** validation, writing – review and editing, **Irfan Ullah:** validation, writing – review and editing, **Raheel Ahmed:** validation, writing – review and editing, **Chadi Alraies:** supervision, validation, writing – review and editing, **Gregg C. Fonarow:** supervision, validation, writing – review and editing.

## Conflicts of Interest

Dr. Fonarow reports consulting for Abbott, Amgen, AstraZeneca, Bayer, Boehringer Ingelheim, Cytokinetics, Eli Lilly, Johnson & Johnson, Medtronic, Merck, Novartis, and Pfizer. The other authors declare no conflicts of interest.

## Supporting information

Supporting File updated.

## Data Availability

All data generated or analyzed during this study are included in this published article and its supporting information files and are freely available on the CDC WONDER database.
