# Supplementary material for: Trends in Cardiovascular Mortality in Patients With Chronic Kidney Disease From 1999 to 2020: A Retrospective Study in the United States
Source: Clin Cardiol. 2025 Jul 31;48(8):e70174. doi: 10.1002/clc.70174 (PMC12312204; doi:10.1002/clc.70174)

**Supplementary File**

**Trends in Cardiovascular Mortality in Patients with Chronic Kidney Disease from 1999 to 2020: A Retrospective Study in the United States**

This file is created by the authors to provide a better understanding of their work.

**Supplementary Table S1:** Trends in Cardiovascular-Related Mortality in patients with Chronic Kidney Disease in the United States from 1999 to 2020, stratified by stage of Chronic Kidney Disease (CKD: chronic kidney disease, AAMR: age-adjusted mortality rate, AAPC: average annual percent change)

| 1999-2010 | | | | 2011-2020 | | | |
| --- | --- | --- | --- | --- | --- | --- | --- |
| Cause of Death | Deaths (n) | AAMR | AAPC | Cause of Death | Deaths (n) | AAMR | AAPC |
|  |  |  |  | CKD 1 | 82 | - | - |
|  |  |  |  | CKD 2 | 732 | 0.02 (0.02 to 0.02) | -4.86 (-11.43 to 4.4) |
|  |  |  |  | CKD 3 | 14,960 | 0.39 (0.38 to 0.39) | 10.68 (5.69 to 17.82) * |
|  |  |  |  | CKD 4 | 13,212 | 0.34 (0.33 to 0.35) | 6.66 (3.46 to 12.17) * |
| End-stage renal disease | 95,586 | 2.65 (2.63 to 2.66) | -0.87 (1.65 to -0.13) | CKD 5 | 105,806 | 2.8 (2.78 to 2.81) | -7.70 (-10.71 to -4.47) * |

*Indicates that the AAPC is significantly different from zero at the alpha = 0.05 level.

**Supplementary Table S2:** State-wise Age-Adjusted Mortality Rates (AAMRs) with their 95% Confidence Intervals for Cardiovascular Mortality Associated with Patients with Chronic Kidney Disease in the United States from 1999 to 2020

| State | Deaths | Age-Adjusted Rate | Age-Adjusted Rate Lower 95% Confidence Interval | Age-Adjusted Rate Upper 95% Confidence Interval |
| --- | --- | --- | --- | --- |
| Alabama | 8694 | 7.561 | 7.401 | 7.721 |
| Alaska | 661 | 7.485 | 6.888 | 8.082 |
| Arizona | 8796 | 5.721 | 5.601 | 5.841 |
| Arkansas | 4556 | 6.266 | 6.083 | 6.449 |
| California | 82131 | 10.292 | 10.221 | 10.363 |
| Colorado | 6608 | 6.692 | 6.53 | 6.855 |
| Connecticut | 6183 | 6.295 | 6.137 | 6.454 |
| Delaware | 1794 | 7.951 | 7.581 | 8.321 |
| District of Columbia | 1561 | 11.771 | 11.183 | 12.36 |
| Florida | 35151 | 6.003 | 5.94 | 6.066 |
| Georgia | 12578 | 6.744 | 6.624 | 6.863 |
| Hawaii | 2971 | 8.255 | 7.955 | 8.554 |
| Idaho | 2464 | 7.177 | 6.892 | 7.461 |
| Illinois | 23452 | 7.775 | 7.675 | 7.875 |
| Indiana | 14824 | 9.679 | 9.522 | 9.835 |
| Iowa | 7343 | 8.259 | 8.067 | 8.45 |
| Kansas | 4771 | 6.556 | 6.368 | 6.743 |
| Kentucky | 8096 | 8.032 | 7.856 | 8.207 |
| Louisiana | 6723 | 6.641 | 6.481 | 6.801 |
| Maine | 2860 | 7.48 | 7.204 | 7.756 |
| Maryland | 13396 | 10.266 | 10.091 | 10.441 |
| Massachusetts | 11658 | 6.62 | 6.498 | 6.741 |
| Michigan | 20936 | 8.403 | 8.288 | 8.517 |
| Minnesota | 11819 | 8.883 | 8.721 | 9.044 |
| Mississippi | 5315 | 7.87 | 7.657 | 8.083 |
| Missouri | 12406 | 8.173 | 8.029 | 8.318 |
| Montana | 1434 | 5.443 | 5.16 | 5.726 |
| Nebraska | 4093 | 8.501 | 8.238 | 8.764 |
| Nevada | 2421 | 4.456 | 4.275 | 4.637 |
| New Hampshire | 2524 | 7.704 | 7.402 | 8.007 |
| New Jersey | 15850 | 7.126 | 7.014 | 7.237 |
| New Mexico | 2494 | 5.309 | 5.099 | 5.519 |
| New York | 34339 | 6.957 | 6.883 | 7.031 |
| North Carolina | 21281 | 9.804 | 9.671 | 9.936 |
| North Dakota | 2127 | 10.726 | 10.262 | 11.189 |
| Ohio | 31354 | 10.526 | 10.409 | 10.643 |
| Oklahoma | 8327 | 9.34 | 9.139 | 9.542 |
| Oregon | 8340 | 8.454 | 8.271 | 8.636 |
| Pennsylvania | 31541 | 8.506 | 8.412 | 8.601 |
| Rhode Island | 2684 | 8.821 | 8.482 | 9.159 |
| South Carolina | 10849 | 9.934 | 9.745 | 10.123 |
| South Dakota | 1938 | 8.368 | 7.991 | 8.745 |
| Tennessee | 14237 | 9.62 | 9.461 | 9.779 |
| Texas | 41458 | 8.764 | 8.679 | 8.85 |
| Utah | 2304 | 5.226 | 5.012 | 5.441 |
| Vermont | 1301 | 7.854 | 7.424 | 8.283 |
| Virginia | 13773 | 7.823 | 7.692 | 7.955 |
| Washington | 13673 | 8.964 | 8.812 | 9.115 |
| West Virginia | 5693 | 11.028 | 10.74 | 11.316 |
| Wisconsin | 12906 | 8.738 | 8.586 | 8.89 |
| Wyoming | 696 | 5.633 | 5.212 | 6.055 |

**Supplementary Figure 1:** Trends in Cardiovascular Mortality Associated with Patients with Chronic Kidney Disease stratified by urbanization in the United States from 1999 to 2020
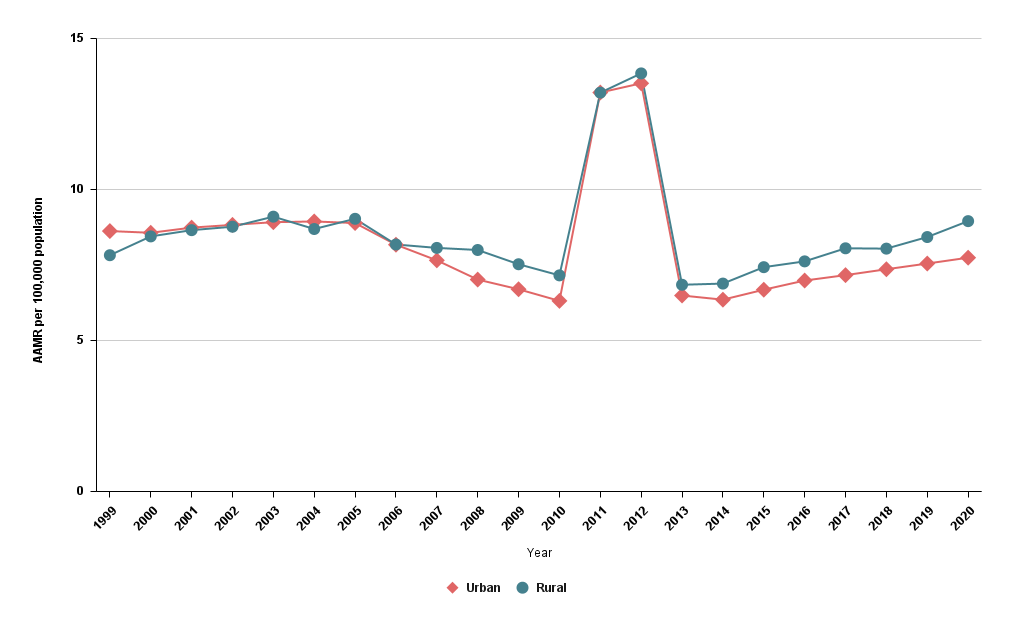


**Supplementary Figure 2:** Trends in Cardiovascular Mortality Associated with Patients with Chronic Kidney Disease stratified by census region in the United States from 1999 to 2020
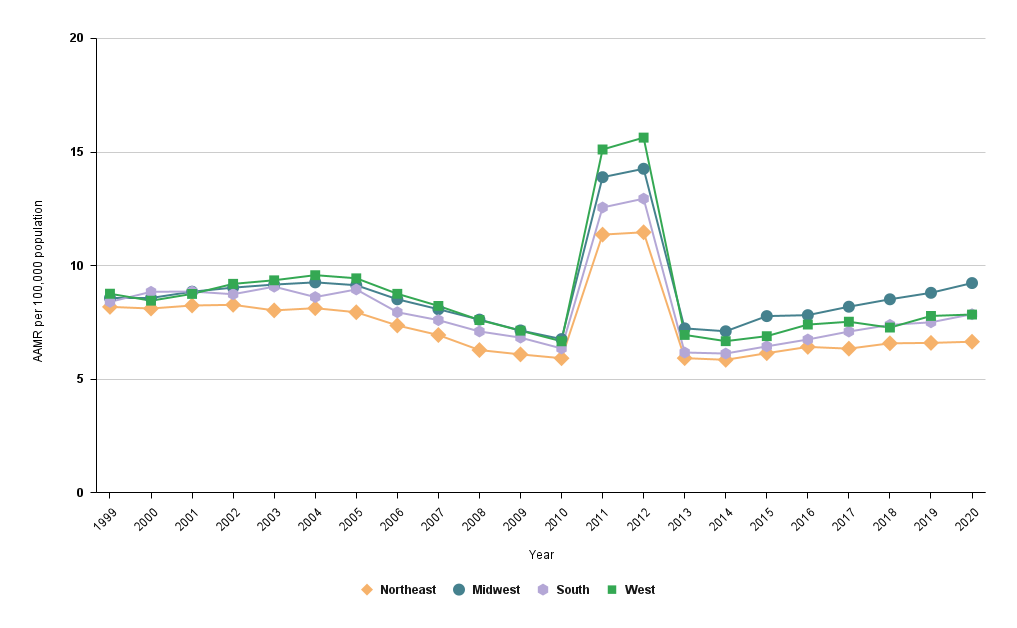


**Supplementary Figure 3:** State-wise deaths and Age-Adjusted Mortality rates (AAMRs) in Cardiovascular Mortality Associated with Patients with Chronic Kidney Disease in the United States from 1999 to 2020
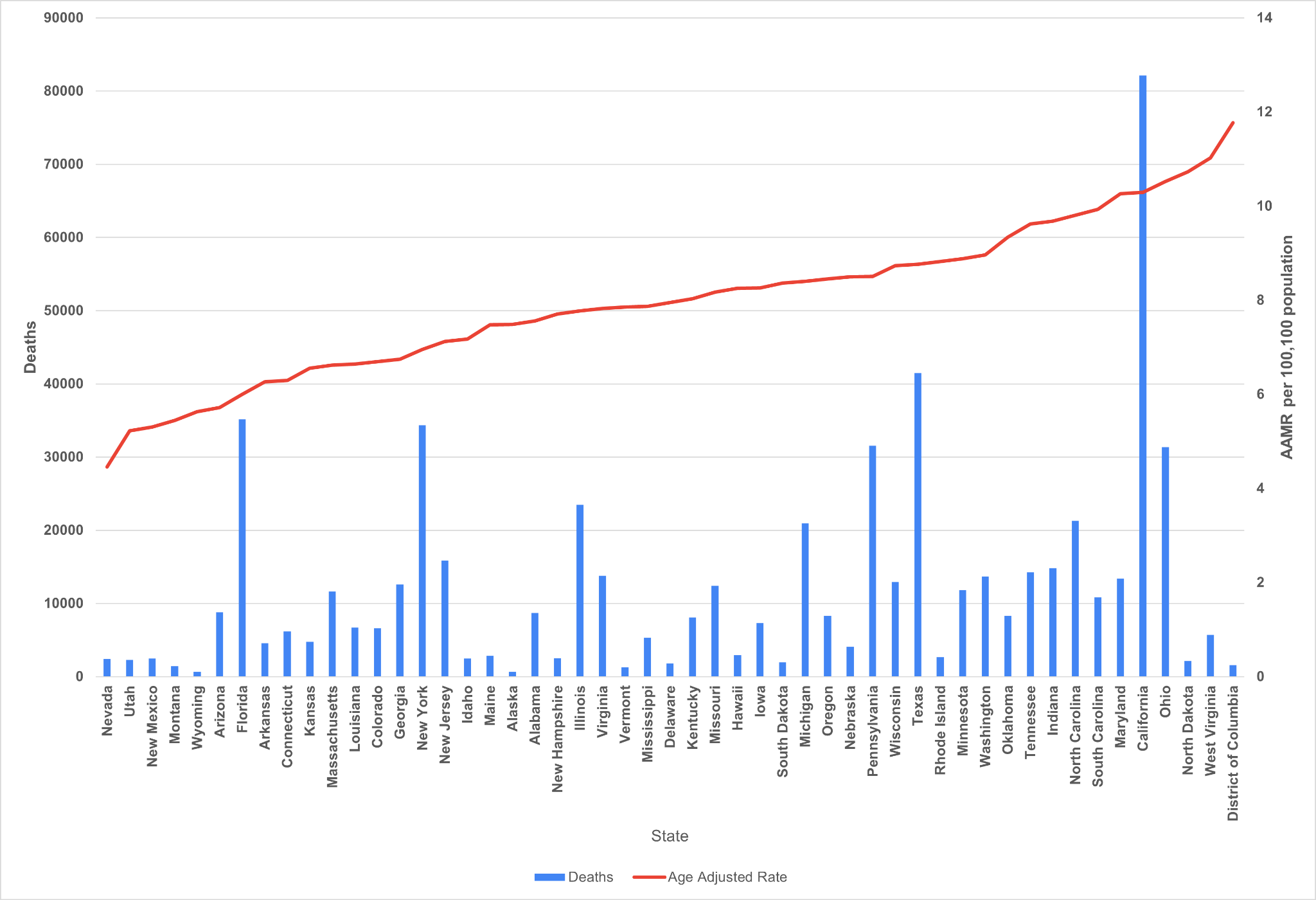

Supplement: Supplementary file 1 — Supporting File updated. [file CLC-48-e70174-s001.docx]
